# Mesenchymal Stem Cell-Conditioned Medium Reduces Disease Severity and Immune Responses in Inflammatory Arthritis

**DOI:** 10.1038/s41598-017-18144-w

**Published:** 2017-12-21

**Authors:** Alasdair G. Kay, Grace Long, George Tyler, Andrei Stefan, Stephen J. Broadfoot, Anna M. Piccinini, Jim Middleton, Oksana Kehoe

**Affiliations:** 1 Biology Department, University of York, Wentworth Way, York, UK; 2 School of Medicine, Keele University, Staffordshire, UK; 3 ISTM at RJAH Orthopaedic Hospital, Keele University, Oswestry, UK; 4 LSSU, Liverpool John Moore’s University, Liverpool, UK; 5 School of Pharmacy, University of Nottingham, Nottingham, UK; 6 Faculty of Health Sciences, School of Oral and Dental Science, University of Bristol, Bristol, UK

## Abstract

We evaluated the therapeutic potential of mesenchymal stem cell-conditioned medium (CM-MSC) as an alternative to cell therapy in an antigen-induced model of arthritis (AIA). Disease severity and cartilage loss were evaluated by histopathological analysis of arthritic knee joints and immunostaining of aggrecan neoepitopes. Cell proliferation was assessed for activated and naïve CD4+ T cells from healthy mice following culture with CM-MSC or co-culture with MSCs. T cell polarization was analysed in CD4+ T cells isolated from spleens and lymph nodes of arthritic mice treated with CM-MSC or MSCs. CM-MSC treatment significantly reduced knee-joint swelling, histopathological signs of AIA, cartilage loss and suppressed TNFα induction. Proliferation of CD4+ cells from spleens of healthy mice was not affected by CM-MSC but reduced when cells were co-cultured with MSCs. In the presence of CM-MSC or MSCs, increases in IL-10 concentration were observed in culture medium. Finally, CD4+ T cells from arthritic mice treated with CM-MSC showed increases in FOXP3 and IL-4 expression and positively affected the Treg:Th17 balance in the tissue. CM-MSC treatment reduces cartilage damage and suppresses immune responses by reducing aggrecan cleavage, enhancing Treg function and adjusting the Treg:Th17 ratio. CM-MSC may provide an effective cell-free therapy for inflammatory arthritis.

## Introduction

There is no cure for Rheumatoid Arthritis (RA) and life expectancy of sufferers may be reduced by up to 18 years^1^. Therapeutic interventions include disease modifying anti-rheumatic drugs (DMARDs) and biologic treatments such as anti-TNFα, anti-IL1, anti-IL6R, anti-CD20 and T-cell co-stimulation blockers. However, 30–58% of patients do not respond to biologics such as anti-TNFα^2–4^, 30–40% lose responsiveness over time^5,6^ and ~50–58% discontinue the therapy within 2 years^3,4,7^. Furthermore, biologic therapies can cause severe side effects including increased risk of infection, hypertension and lymphoma^1^, are expensive and require continuous subcutaneous injections^7^. There is therefore a need for efficacious, safer and affordable therapeutics.

Alternative treatments include stem-cell therapy. Mesenchymal stem cells (MSCs) exert immunomodulatory functions, including inhibition of T cell proliferation, interference with B cell function and dendritic cell maturation and promotion of anti-inflammatory macrophage-mediated responses^8^. Although stem-cell therapy presents a promising alternative treatment, questions remain over differentiation of stem cells where tissue regeneration is not the primary goal. Moreover, autologously sourced MSCs must be harvested from patients and cultured *in vitro* to achieve therapeutic cell numbers.

We previously demonstrated that MSCs reduce inflammation in a murine antigen-induced arthritis (AIA) model^9^. MSCs respond to the inflammatory environment by enhancing expression of immunosuppressive factors thereby influencing target cells through paracrine mechanisms^10^. This involves the production of signalling molecules such as TGF-β1, IL-10, CCL9, IFN-α, IFN-β, nitric oxide (NO), VEGF, FGF, HGF, PDGF and membrane-bound vesicles, including microvesicles and exosomes^11^. We therefore hypothesised that these soluble factors, which are present in serum-free MSC-conditioned medium (CM-MSC)^12–19^, may be responsible for the therapeutic effects of MSCs^12–15^.

Similarly to MSCs, CM-MSC can be therapeutically administered. Thus, here, we tested the therapeutic potential of CM-MSC in the AIA model of inflammatory arthritis. The effects of CM-MSC therapy were directly compared to those of MSC therapy through assessment of histological outcomes, TNF-α production and cartilage loss. The immunomodulatory action of CM-MSC was investigated through examination of T cell activation, differentiation and proliferation, and quantification of immunomodulatory factors. We propose CM-MSC as a potential therapeutic approach for the treatment of inflammatory arthritis.

## Results

### CM-MSC ameliorates severity of inflammatory arthritis

AIA is a well-established acute model of inflammatory arthritis that mimics many clinical and histopathological changes seen in human RA^20–23^.

**Figure 1:**
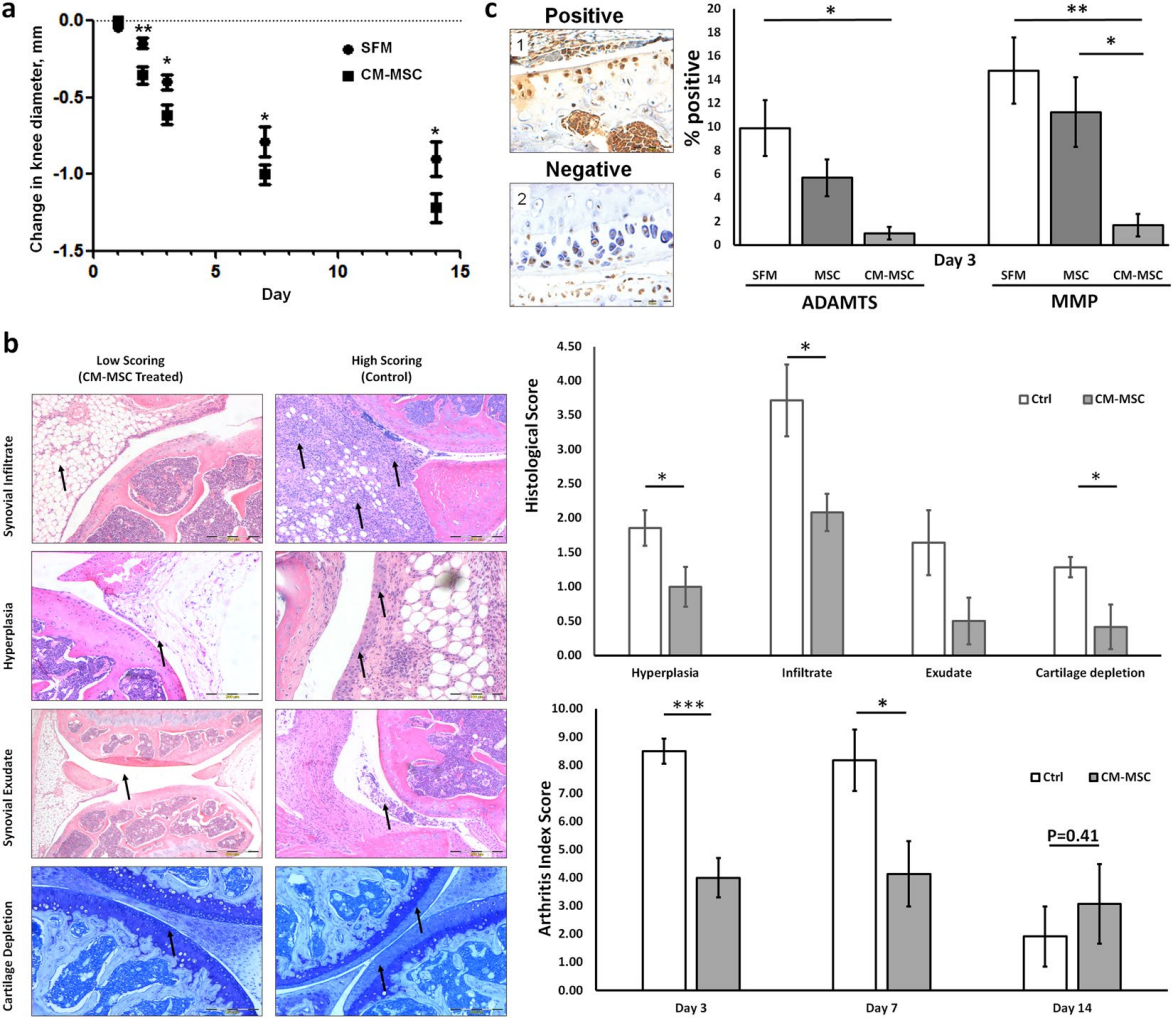
Effects of intra-articular injections of CM-MSC in AIA. (**a**) Knee diameter (mm) as an index of swelling (joint inflammation) measured at days 1, 2, 3, 7 and 14 after arthritis induction. Significant reductions are seen following CM-MSC injection in AIA mice (n = 21 (day 1 & 2), 16 (day 3), 12 (day 7), 6 (day 14) mice per group). (**b**) Histopathological symptoms of AIA used to assess disease severity. Representative images for low and high scoring taken from CM-MSC treated joints and control SFM treated joints respectively. Arrows show areas of interest. Data indicates CM-MSC prompts reductions in synovial infiltrate (leukocyte accumulation in the synovium), hyperplasia of the synovial intima and cartilage depletion (p < 0.05) at day 3 post-arthritis induction. Arthritic Index is reduced in CM-MSC treated mice at days 3 and 7 post-arthritis induction (p < 0.001 and p < 0.05 respectively) with no difference detected at day 14 (p = 0.41). (**c**) Cells involved in aggrecan cleavage due to ADAMTS5 and MMP activity (%). CM-MSC significantly reduces aggrecan breaks due to ADAMTS5 and MMPs. Representative positive DIPEN staining (c1) shows extracellular matrix staining, which is absent in control stains (c2) (scale bars = 200 µm) (*p < 0.05; **p < 0.01; ***p < 0.001).

CM-MSC treatment reduced joint swelling as a measure of inflammation compared to SFM control at days 2 (p < 0.01), 3 (p < 0.05), 7 (p < 0.05) and 14 (p < 0.05) post-arthritis induction (2 way ANOVA with Bonferroni post-hoc) (Fig. 1a, Table S1). Significant reductions were also recorded in synovial infiltrate, hyperplasia of the synovial intima and cartilage loss (p < 0.05) at day 3 following CM-MSC treatment and in overall arthritis index at 3 days and 7 days post-arthritis induction (p < 0.001, p < 0.05 respectively) (Mann Whitney) (Fig. 1b). By day 14, knee sections displayed signs of recovery and all histological scores were reduced in control and treated animals, giving no significant difference between control and test arthritis index at this time. Overall, these results indicate that CM-MSC treatment significantly reduces disease severity and acute cartilage damage in AIA.

### CM-MSC therapy reduces aggrecan breakdown

To further examine the mechanism underlying reduction in cartilage loss, we assessed the presence of the neoepitopes …NVTEGE and …VDIPEN generated during the enzymatic cleavage of aggrecan 3 days after arthritis induction. Compared to MSC treatment, CM-MSC showed a higher trend in the reduction of aggrecan cleavage in both early, ADAMTS-driven, and late, MMP-driven degradation (CM-MSC p < 0.05, p < 0.001 respectively, MSC p < 0.05 for both, 2-way ANOVA) (Fig. 1C, Table S2) with clearly visible regions of extracellular staining of aggrecan neoepitopes occurring in untreated cartilage that are not visible in CM-MSC joints. No differences were found between CM-MSC and MSC treatments at any time point (p > 0.05). Together, these data indicate that CM-MSC and MSC treatments have similar therapeutic efficacy in AIA.

### CM-MSC treatment prevents TNFα rise in serum from arthritic mice

**Figure 2:**
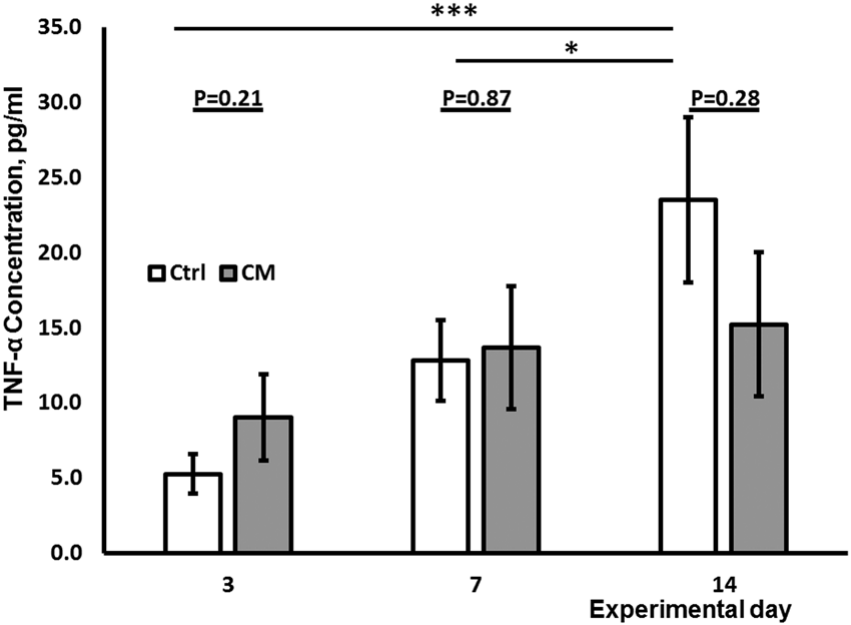
CM-MSC suppresses TNF-α in serum. ELISA of circulating TNF-α in blood serum measured at 3, 7 and 14 days post arthritis induction. CM-MSC suppresses time-dependant increases in TNF-α observed in control animals (n = 6 mice per group) but does not statistically differ from control for each timepoint. (*p < 0.05; ***p < 0.001).

TNFα is a key driving cytokine in the pathogenesis of RA. To determine whether amelioration of AIA following CM-MSC treatment was associated with TNFα blockade or antagonism, we measured changes in TNFα levels in serum of arthritic mice. As expected, SFM control mice showed increasing TNFα levels in serum from day 3 to 14 and 7 to 14 post-arthritis induction (Fig. 2, Table S3) (p < 0.001, p < 0.05 respectively, 2 way ANOVA, n = 6). CM-MSC treatment prevented these TNFα increases observed over time although there were no statistically significant differences found between levels of TNFα in serum of CM-MSC treated or SFM treated animals.

### CM-MSC has no effect on T cell proliferation

**Figure 3:**
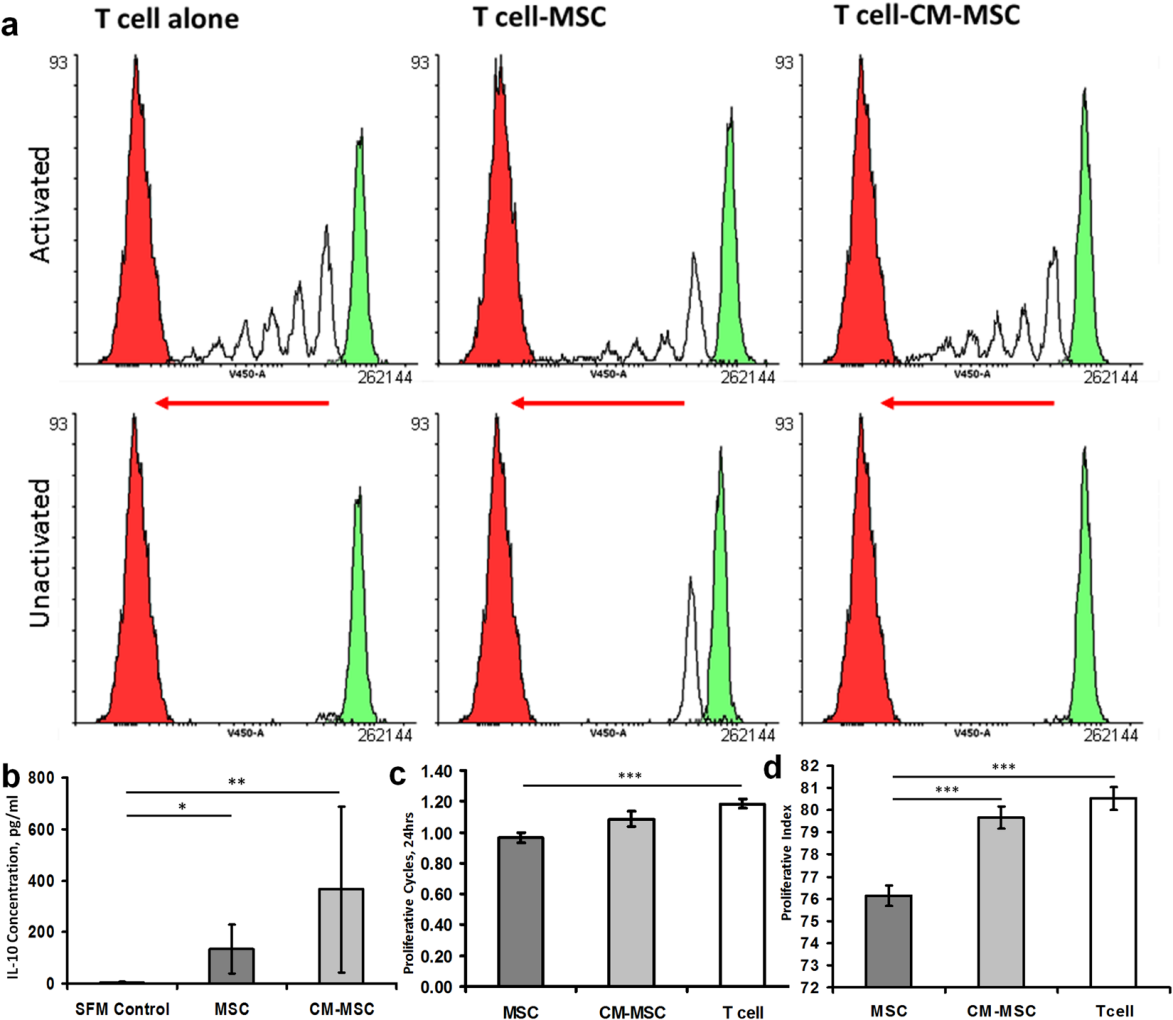
Decreased T cell proliferation in co-culture with MSC assessed using Violet Proliferation Dye 450 (VPD450) (**a**) Proliferative cycle representative images show individual colourless peaks with a green peak representing initial T cell population at day 1 and red peaks demonstrate unstained T cell signal intensities as negative control. MSC/T cell co-culture with activation reduces peak frequency and height. (**b**) ELISA of IL-10 in conditioned medium from T cell culture shows increased concentration of IL-10 compared to fresh serum-free RPMI medium control (n = 4). (**c**) Proliferative cycles per 24 hours culture (population doubling frequency) and (**d**) proliferative capacity (representative of proportion of cells undergoing proliferation) are significantly reduced following MSC co-culture (n = 4 spleens with 2 replicates for each) (*p < 0.05, ***p < 0.001).

We next investigated whether the therapeutic effect observed upon treatment with CM-MSC or MSCs was due to changes to T cell function, which plays a central role in RA. T cell proliferation was assessed for percentage of non-dividing cells, peaks of gradual division (proliferative index)^24^ representative of the proportion of cells undergoing division accounting for population undergoing varied total cycles of division (e.g. accounting for cells that undergo less/more than the average number of population doublings), and 24 hour proliferative cycles (population doublings)^25^ for T cells isolated from spleens of healthy mice following *in vitro* activation. T cells do not proliferate in culture, unless activated with anti-CD3/CD28, and undergo cell death in absence of IL-2, which is produced *in vivo* by activated T cells^26^. Proliferative cycles were assessed using Violet Proliferation Dye (VPD450) observing peaks for each proliferative cycle completed (Fig. 3a).

To investigate inhibition of T cell activation in co-culture with MSCs, we measured IL-10 in CM-MSC and cell supernatant collected after 5 day of co-culture. When comparisons were made with serum-free RPMI 1640 used in controls, a significant increase in IL-10 was observed in medium of T cells following co-culture with MSC (p < 0.05) and CM-MSC (p < 0.01) (Fig. 3b, Table S5) (1 Way ANOVA with Dunn’s post-hoc). We did not detect IL-10 in CM-MSC prior to application to the cells. These data indicate that, similarly to MSCs, CM-MSC increases IL-10 production in co-culture with activated T cells.

The presence of MSCs in co-culture reduced the number of proliferative cycles (p < 0.001, 1-way ANOVA with Tukey’s post-hoc, n = 11) (Fig. 3c) and proliferative index in comparison to T cells alone and T cells with CM-MSC (p < 0.001, 1-way ANOVA with Tukey’s post-hoc) (Fig. 3d). No difference was found between CM-MSC and T cells alone (Table S4).

### CM-MSC enhances FOXP3 expression in spleens and lymph nodes

**Figure 4:**
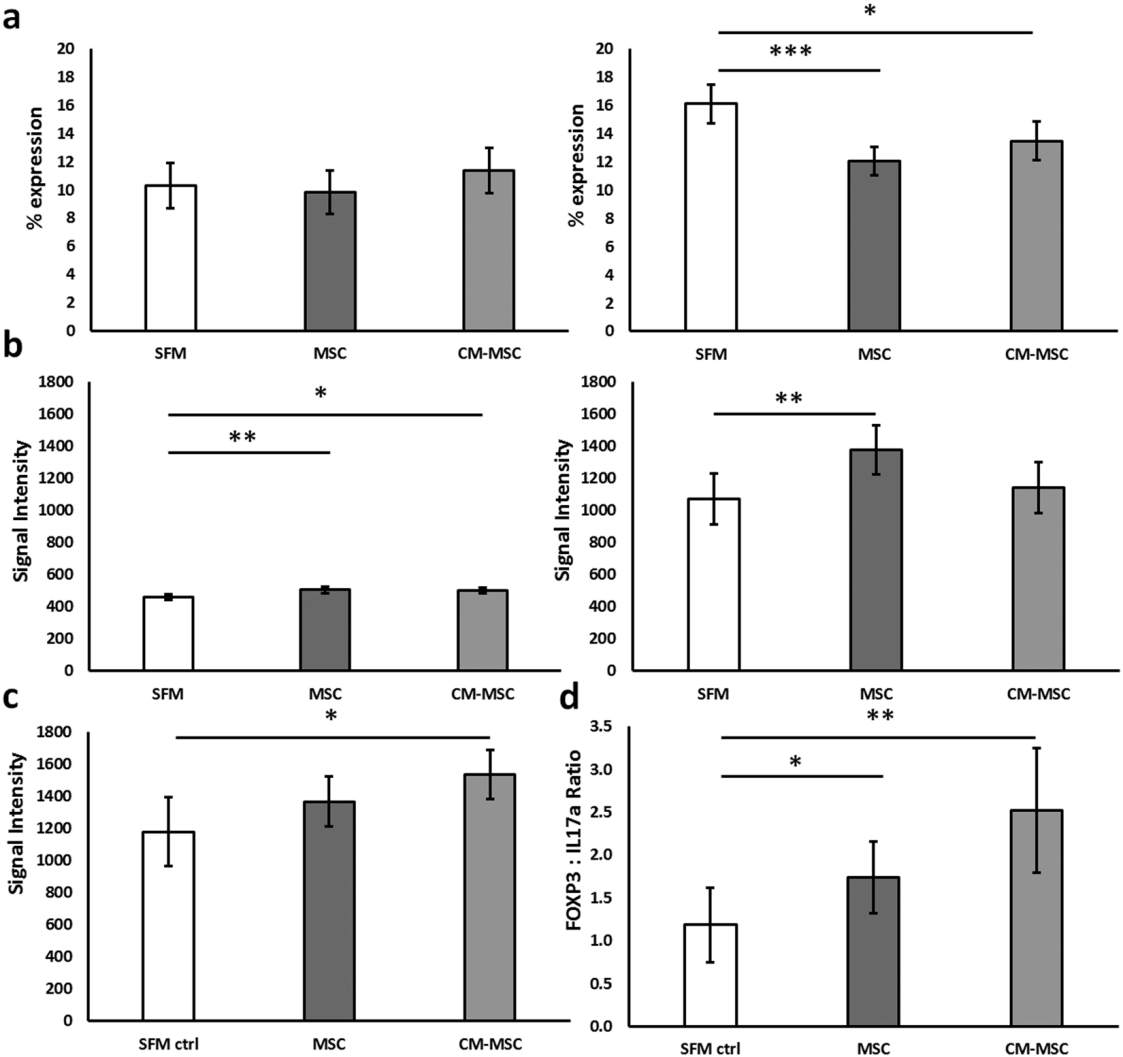
Increased immunosuppressive/anti-inflammatory T cell subsets following CM-MSC treatment at 3 days post induction of arthritis. (**a**) Surface staining of CD4 assessed proportion of CD4+ cells in spleens (left) and lymph nodes (right). (**b**) Intracellular staining for key cytokines characteristic of Treg (FOXP3) in spleens (left) and lymph nodes (right) and (**c**) Th2 (IL4) following 4 hours culture with inhibition of protein transport using Brefeldin A for T cells from CM-MSC and MSC treated mice and SFM treated controls (mean fluorescence intensity (MFI)). (**d**) The ratio of percentage FOXP3+ to IL17a+ cells (Treg:Th17) was calculated showing an improved ratio of FOXP3:IL17a expression following CM-MSC co-culture. All data were obtained from AIA mice at day 3 post arthritis induction. (*p < 0.05; **p < 0.01; ***p < 0.001).

Increased production of IL-10 during T cell co-culture with MSC or CM-MSC led us to investigate the polarisation of naïve T cells into effector lineages with immunosuppressive/anti-inflammatory function following CM-MSC or MSC treatment. FOXP3 is a key transcriptional regulator for Treg development and function, with increased expression conferring enhanced immunosuppressive properties^27^. Tregs isolated from spleens and lymph nodes in close proximity to the treated joint were analysed for CD4+ CD25+ FOXP3+ positivity. In the spleen, 3 days post arthritis induction, the mean percentage of CD4+ cells did not vary between MSC, CM-MSC and SFM control treatments (9.83 ± 1.54%, 11.36 ± 1.60%, 10.30 ± 1.60% respectively). However, lymph nodes showed significantly decreased numbers of CD4+ cells in both MSC and CM-MSC treatments (12.06 ± 0.99%, p < 0.001 and 13.49 ± 1.39%, p < 0.05, respectively) compared to SFM controls (16.11 ± 1.38%)) (ANCOVA with Bonferroni post-hoc) [Fig. 4a]. No differences were observed between CM-MSC and MSC treatments.

The cellular expression of FOXP3 increased upon both MSC (Mean fluorescence intensity (MFI) 504.4 ± 18.3, p < 0.001) and CM-MSC (MFI 500.6 ± 18.1, p < 0.05) treatments at day 3 compared to controls (MFI 459.6 ± 18.3) in spleens of AIA mice and upon MSC treatment (MFI 1048.2 ± 88.3, p < 0.05) compared to controls (MFI 813.5 ± 123.1) in lymph nodes (ANCOVA with Bonferroni post-hoc) [Fig. 4b]. CM-MSC showed a higher tendency to increase Treg numbers in the spleen compared to MSC treatment (normalised per mg tissue; MSC: 4249 ± 2930; CM-MSC: 5273 ± 3852; control: 3832 ± 2872; p > 0.05, 1 Way ANOVA, n = 6). These data show that CM-MSC treatment of arthritic mice enhances FOXP3 expression in CD4+ T cells.

### Increased production of IL-4 and FOXP3 and restored Treg:Th17 balance following CM-MSC treatment

The pathogenesis of RA is characterized by an imbalance between anti-inflammatory (Th2, Treg) and pro-inflammatory (Th1, Th17) T cells with decreased Treg and increased Th17 cell numbers^28^. To further investigate T cell differentiation, intracellular staining was performed to establish the expression of IL-4 (Th2) and IFN-γ (Th1) and the ratio of FOXP3 (Treg) to IL-17a (Th17) expressing cells in splenocytes from arthritic mice following MSC or CM-MSC treatment.

IL-4 expression increased significantly following CM-MSC treatment (Fig. 4c), indicating that CM-MSC enhances Th2 cell immunosuppressive activity. Although IL-4 expression was not significantly increased following MSC treatment, the proportion of IL-4 expressing cells (Th2) (27.33 ± 4.64%) significantly increased compared to CM-MSC (20.77 ± 3.42%) and control (20.97 ± 2.53%) (p < 0.05, 1-way ANOVA with Tukey’s post-hoc, n = 12). T cell expression of IFN-γ was not affected by CM-MSC or MSC.

CM-MSC treatment also decreased the proportion of pro-inflammatory Th17 cells (20.99 ± 4.93%) compared to MSC treatment (30.55 ± 6.37%) (p < 0.05, 1-way ANOVA with Tukey’s post-hoc, n = 12) and control (23.39 ± 5.18%). Furthermore, the ratio of FOXP3:IL17a expressing cells significantly increased following CM-MSC treatment (2.52 ± 1.80) compared to SFM control (1.18 ± 0.75; p < 0.01) and MSC treatment (1.74 ± 1.32; p < 0.05; Kruskall-Wallis with Dunn’s post-hoc, n = 12) (Fig. 4d). Thus, CM-MSC promotes immunosuppressive/anti-inflammatory T cell activity and restores the Treg/Th17 balance in AIA.

## Discussion

We previously demonstrated that MSC therapy in AIA reduces inflammation, leukocyte (predominantly neutrophil) exudate into the joint cavity and disease severity^9^. However, MSC therapy requires surgery to harvest autologous cells and differentiation of transplanted cells is difficult to control. Such limitations restrict translation of stem cell therapies into clinical use. Stem cell-based, cell-free therapies bypass these issues and allow banking and standardisation of treatments, facilitating rapid clinical translation. Here, we evaluated the therapeutic potential of cell-free CM-MSC as a novel strategy for the treatment of inflammatory arthritis and test the hypothesis that therapeutic MSCs act in a paracrine manner. Our *ex-vivo* and *in-vivo* studies provide evidence for CM-MSC acting as a therapeutic agent by significantly reducing the histopathological signs of inflammation and joint damage characteristic of arthritis, including cartilage breakdown. We demonstrate that CM-MSC exerts its immunosuppressive/anti-inflammatory function by increasing FOXP3 expression in Tregs and improving Treg:Th17 ratios. CM-MSC treatment not only closely recapitulates the effects seen upon MSC treatment^9^, but also eliminates the risks linked to cell transplants.

CM-MSC treatment reduced cartilage destruction in AIA mice. We investigated aggrecan neoepitopes formation through enzymatic cleavage by ADAMTS and MMPs. RA is a disease characterised by progressive joint destruction. The enzymatic cleavage of aggrecan is a key event in the development of RA^29^ and is due to the action of proteolytic enzymes directed by inflammatory mediators such as cytokines, prostaglandins, matrix degradation products, complement and growth factors^30^. Moreover, RA is characterised by the formation of a pannus, accumulated rheumatoid synovial tissue that invades cartilage and bone. However, cartilage breakdown occurs in RA even in the absence of pannus formation^31^. Matrix metalloproteinases (MMPs), predominantly MMP’s −1, −3 and −13^32^, and aggrecanases, in particular aggrecanase 1 (ADAMTS4) and aggrecanase 2 (ADAMTS5)^33–35^, are responsible for the breakdown of cartilage and release of peptide fragments, termed neoepitopes, into the rheumatoid joint. RA sufferers have been shown to have reduced intact aggrecan, but increased aggrecan fragments in circulation^36^. Citrullinated epitopes of degraded proteoglycans from cartilage have been detected in >60% of RA sufferers and implicated in increased pro-inflammatory cytokine production in clinical patients^37–40^. Furthermore, stimulation of peripheral blood mononuclear cells of RA patients with citrullinated aggrecan neoepitopes leads to a pro-inflammatory response characterised by T cell proliferation and increased formation of IL17a expressing Th17 cells not observed with PBMC of healthy individuals^39^. The inflammatory response to citrullinated self-antigens has been shown to increase over responses to wild type antigens in mice^41^ and to native epitopes in humans^42^. Anti-citrullinated protein antibodies occur with higher specificity (~96%) in RA patient serum over rheumatoid factor^43^. Autoimmunity may, in fact, develop in RA largely due to extracellular fragments such as aggrecan neoepitopes forming MHCII complexes with cells that fail to be negatively selected during thymic development. For example, aggrecan specific B cells have been shown to readily acquire aggrecan neoepitopes and function in antigen presenting to CD4+ T cells leading to T cell activation and effector T cell formation^44^. Consequently, autoantigen release, in the form of aggrecan neoepitopes from inflamed cartilage where joint destruction is present, may promote RA pathology even in the absence of pannus formation. MMP activation occurs in response to cytokine signalling, in particular pro-inflammatory cytokines IL-1β and TNF-α, which are key therapeutic targets in the control of RA using biologic treatments. An awareness of effects of treatment methodologies on the breakdown of aggrecan is vital in the development of effective novel therapeutics.

We assessed …NVTEGE (ADAMTS-cleaved) and …VIPEN (MMP-cleaved) aggrecan neoepitope formation in mouse knee-sections following CM-MSC treatment. Early cartilage destruction in AIA is mediated by the ADAMTS family of enzymes^15,19^ and loss of the ADAMTS5 gene has been shown to prevent cartilage destruction in mouse models of arthritis^11^. CM-MSC treatment reduced aggrecan degradation by both ADAMTS and MMPs. CM-MSC therapy provided a more beneficial effect than MSC treatment in the time period examined, potentially due to the need for MSCs to home and adjust to their environment. Our results in mice support previous studies examining neoepitope formation due to MMP and aggrecanase activity in humans, with no co-ordinated activity of the enzymes and regional correlation between cartilage damage and epitope formation, mainly in the superficial layer of cartilage^45^. Our results demonstrate potentially beneficial reductions in neoepitope formation following CM-MSC treatment which may therefore exacerbate the progression of RA through a resolution of citrullinated aggrecan neoepitope formation.

Following CM-MSC treatment, we did not observe a reduction in circulating TNF-α as seen upon MSC treatment but over time increases in circulating TNF-α were observed in untreated AIA mice. CM-MSC suppressed the increase in TNF-α observed in untreated AIA mice. We hypothesised that soluble factors released by MSCs reduce endothelial permeability, decreasing leukocyte accumulation in the synovial fluid of inflamed joints^9^.

Our results showed suppressed proliferation of T cells when co-cultured with MSC but not in the presence of CM-MSC alone. There is conflicting reports as to the efficacy of applying soluble molecules secreted by MSC as immunomodulatory therapy and some debate as to the mechanism that elicits suppression of T cell proliferation. In general, reports confirm the involvement of soluble hepatocyte growth factor (HGF) which induces IL-10 production by monocytes^46^, indoleamine 2,3-dioxygenase (IDO), transforming growth factor (TGF-β1) and prostaglandin E2 (PGE2)^46–51^. A majority of reports of successful suppression of T cell proliferation using soluble factors secreted by MSC utilise either transwell systems^52–56^ or similar methods to inhibit cell contact whilst maintaining MSC presence in the system e.g. encapsulated cells^57^. Additionally, these studies predominantly measure the proliferation of T cells in either mixed lymphocyte cultures^52–54,56^ or in mixed splenocytes^55^. The interaction of MSCs with CD8+ effector T cells, B cells, Natural Killer cells and dendritic cells are well described^58–60^ and consequently the mechanisms at work in mixed lymphocyte cultures may be mediated through MSC influences on CD4− cells that subsequently act upon CD4+ T cells. Studies reporting the need for cell contact to exhibit a proliferative effect commonly utilise cell supernatants^61^ or removal of MSCs from co-culture prior to measurement, and application of enriched T cells rather than mixed lymphocyte populations^62–64^. These variations in study methodology make it difficult to ascertain the precise mechanisms involved in suppression of T cell proliferation though it seems likely that, whilst CM-MSC conveys reduced potency in comparison to the influence of MSC in the system, the inclusion of mixed population lymphocytes may restore similar immunomodulatory properties of CM-MSC^52–56^.

In this study, T cells were cultured with CM-MSC and activated with a CD3ɛ/CD28 activation beads (Miltenyi) at the time of seeding T cells into MSC plated wells. T cells activated with cognate antigen commence proliferation after approximately 24 hours^62^, so results utilising single-dose treatment with CM-MSC could potentially reflect a depletion of the required suppressive stimuli prior to the initiation of T cell division. In the AIA murine model of inflammatory arthritis, T cell proliferation is initiated with antigen challenge and treatments are administered 24 hours post-induction. Consequently, *in vivo* T cell proliferation would be underway in this model at the time of MSC or CM-MSC intervention. The anti-proliferative capacity of secreted factors from MSC may therefore be applied here in a timely manner to elicit a positive therapeutic response, not ruling out the possibility that treatment with CM-MSC is inducing suppression of T cell proliferation *in vivo* even when suppressive effects are not observed for CM-MSC *in vitro*.

Our findings are consistent with previous studies that suggest cell supernatants require increased concentrations compared to MSC numbers used in co-culture^63–65^. Similarly, our findings confirmed that the presence of MSC maintains T cell survival in the absence of CD3ɛ/CD28 activation consistent with prior studies^63^. MSCs of bone marrow origin exert suppressive effects through arrest of lymphocyte cell cycle^66,67^, apoptosis of T cells^67,68^ or induction of T cell suppressive dendritic cells that induce Treg production and enhance IL-10 secretion^69,70^. Interactions are, however, dependent on MSC dose concentration^66^. Some evidence suggests that suppression seen with higher concentration CM-MSC may act via cell apoptosis whilst transwell studies commonly describe T cell cycle arrest^67,68^. With direct cell contact, low dose MSC are sufficient to suppress T cell proliferation whilst CM-MSC alone requires increased equivalent MSC:T cell concentrations to exhibit suppressive effects^64,65,67,68^. In this study, proportionally equivalent dosage of CM-MSC (to match MSC secretions for 5.0 × 10^5^ cells over a 48 hour period) was assessed for T cell suppression to match MSC concentration for *in vivo* experimentation. CM-MSC was not sufficiently potent to induce T cell suppression. This result coupled with the positive histological improvements suggest that suppression of T cell proliferation may not be the key mechanism for improved outcomes following treatment in AIA, however the timing of treatment administration cannot definitively exclude this possibility. A further study examining dose repeats and timing of application in co-culture for CM-MSC and CD4+ T cells may further elucidate the mechanisms involved.

As well as inhibiting proliferation of T cells, MSCs increase Treg numbers and IL-4 and IL-10 expression whilst reducing IL-6 and IL-17 secretion and Th1 and Th17 differentiation^71–75^. Our data show a similar decrease in CD4+ T cell numbers in spleen and lymph nodes of arthritic mice upon CM-MSC and MSC treatment, suggesting that CM-MSC is able to convey a response *in vivo* similar to that of MSC. Our study also shows increased Treg numbers upon CM-MSC and MSC treatment and demonstrates that CM-MSC can increase IL-4-expressing Th2 cells and FOXP3 expression by activated T cells. The concentration of IL-10 increased in medium of T cells co-cultured with MSC or in CM-MSC. The origin of this IL-10 was not assessed and so may be due to production by MSCs or T cells. The increased presence of FOXP3 expressing Tregs would likely be associated with an increased presence of IL-10 in medium. Furthermore, the soluble secretions of MSC in the presence of activated T cells show enhanced IL-10 secretion compared to MSC in standard culture^63^. These results are in line with work done by others showing that mesenchymal cell-derived factors skew T cell polarization toward Treg and reduce pro-inflammatory cytokine expression^76^. Previous reports suggest immunosuppressive effects of MSC function through the enhanced activity and development of FOXP3 expressing T cells and not a reduction in pro-inflammatory IFN-γ expressing Th1 cells^63^.

This study is an early demonstration of CM-MSC induced increases in Treg induction and expression of FOXP3, alongside positive adjustment of the Treg:Th17 balance, without Th17 or Th2 cell induction at 3 days post-arthritis induction in AIA mice. In RA, Treg differentiation is inhibited causing the ratio of Tregs to Th17 cells to be disrupted^77^. Our study suggests a therapeutic mechanism for CM-MSC that counteracts this effect.

MSCs stimulated by IL17a function to suppress T cell proliferation (as observed in our co-culture experiments), IFN-γ, TNF-α and IL-2 from Th1 cells and induce Tregs^15^. IL-17a overexpression accelerates the development and severity of synovial inflammation and bone erosion in inflammatory arthritis^78^. We have shown a shift to restore the homeostatic balance of Th17 to Treg cells. Further investigation will include an *in vivo* examination of T cell differentiation using immunohistochemical staining of knee sections to investigate the expression of FOXP3 and IL17a in tissue in the mouse joint. We hypothesise that these results will provide evidence that the *in vitro* findings translate to a similar restorative shift in T cell development within the mouse following treatment with MSC or CM-MSC. In addition to this, we aim to evaluate the function outcomes of the T cell immunomodulation observed by culturing naïve CD4+ T cells isolated from spleens of AIA mice following treatment to evaluate their interactions with effector cells. We hypothesise here that the response to cells from CM-MSC treated mice will result in reduced activation and proliferation of immune effector cells comparative to control, untreated animals. This can be evaluated both *in vitro* using cells isolated from spleens of AIA mice undergoing treatment and *in vivo* using the current assay technique with immune cell status being assessed using immunohistochemistry on joint sections.

We previously showed that MSC therapy reduces the concentration of circulating TNFα which would facilitate the restoration of homeostatic T cell differentiation^9^. We evaluated the differentiation of naïve CD4+ T cells to quantify Th1 and Th2 cells and the ratio of Tregs:Th17. CM-MSC, but not MSCs, restored the ratio of Tregs:Th17 cells from a diseased state (ratio ~1:1 in SFM controls) to a homeostatic level (ratio ~3:1 in CM-MSC treated animals). The reduced polarisation of IL-17a secreting Th17 cells demonstrated here supports our previous study demonstrating reduced neutrophil accumulation in the joints of MSC treated mice^9^.

Further work is needed to clarify the prolonged effects of CM-MSC treatment, dose requirements and frequency of treatment to achieve optimal therapeutic outcomes. The potential exists to further refine CM-MSC by removing, for instance, waste products of cell metabolism. Genomic and proteomic analysis of CM-MSC will identify the anti-inflammatory component(s) of CM-MSC that could be purified and used in isolation or in combination to maximise the therapeutic response. Although one of the challenges ahead is to identify the exact mechanism of action of CM-MSC *in vivo*, CM-MSC represents an attractive therapeutic with potential for inflammatory arthritis treatment.

This study demonstrates the potential of CM-MSC as a therapy for inflammatory arthritis. Increased expression of anti-inflammatory, and suppression of pro-inflammatory, cytokines and a reduction in aggrecan breaks can be seen to ameliorate the symptoms of inflammatory arthritis in the AIA model. The exclusion of living cells from a cell-based therapy offers several advantages in clinical translation. Treatments can be assessed, measured and standardised to ensure reliability and reproducibility. Regulatory restrictions call for tight controls on the introduction of living cells from donor to recipient, and these regulations will be more readily met by a “cell-free” therapy. The potential for CM-MSC to provide a broad therapeutic strategy for autoimmune inflammatory conditions is significant.

## Methods

### Antigen-induced arthritis (AIA)

Animal procedures were undertaken in accordance with Home Office project licence PPL40/3594. AIA was induced in male C57Bl/6 mice (7–8 weeks) as previously described^79^. Swelling was assessed by measuring the difference in diameter between the arthritic (right) and non-arthritic (left) knee joints (in mm) using a digital micrometer (Kroeplin GmbH).

### Cells

Murine MSCs isolated from BALB/C mice and cryopreserved at early passage (P3-P5)^9^ were thawed and cultured in Iscove Modified Dulbecco Medium (IMDM) (Gibco, UK) supplemented with 9% foetal bovine serum (Gibco, UK), 9% horse serum (Gibco, UK) and 1% penicillin-streptomycin (Cell Expansion Medium, CEM). Cells (P3-P5) were previously characterised as MSC through immunophenotyping of surface markers with flow cytometry and tri-lineage differentiation^9^.

### Production of CM-MSC

MSCs from the same batch used for cell treatment (P3-P5) were seeded in T75 flasks with CEM (~1.3 × 10^4^ cells/cm^2^). At 80–90% confluence, cells were washed with PBS and then serum-free IMDM media (SF-IMDM). Flasks were incubated for 48 hours with 12 ml SF-IMDM at 37 °C, 5% CO_2_. SF-IMDM with no cells was used as a control (SFM). After 48 hours, medium was removed and centrifuged for 5 minutes at 1200 × g to remove cell debris. 11 ml of supernatant was transferred to an Amicon Ultra 15 filter (3 kDa cut-off membrane) and centrifuged at 4000 × g for 40 minutes at 4 °C. Filters were flushed repeatedly with supernatant and concentrated CM-MSC was stored at −80 °C^80^. Average cell count following removal of CM-MSC for concentration was 2.8 × 10^6^ cells.

### Intra-articular injection of MSC and CM-MSC

Treatments were 15 μl concentrated CM-MSC or 5.0 × 10^5^ MSC (P4-P5) resuspended in 15 μl of SF-IMDM. 15 μl of concentrated CM-MSC corresponded to cell secretions from 5.08 × 10^5^ cells on average. Treatments or SFM control were injected intra-articularly 1 day post arthritis induction with 0.5 ml monoject (29 G) insulin syringes (BD Micro-Fine, Franklyn Lakes, USA) through the patellar ligament into the right knee joint. Joint diameters were measured at 2, 3, 7 and 14 days post injection. Blood, joints, spleen, inguinal and popliteal lymph nodes were collected immediately post-mortem. Three independent experiments were performed. All measures were taken to reduce animal numbers (n = 5–11 per time point).

### Arthritis Index

Animals were sacrificed for histological analysis at days 3, 7 and 14. Joints were fixed in 10% neutral buffered formal saline and decalcified in formic acid for 4 days at 4 °C before paraffin embedding. Sections (5μm) were stained with haematoxylin and eosin (H&E) or toluidine blue and mounted in Hydromount (National Diagnostics) as described previously^9^. H&E sections were scored for hyperplasia of the synovial intima (0 = normal to 3 = severe), cellular exudate (0 = normal to 3 = severe) and synovial infiltrate (0 = normal to 5 = severe); and Toluidine Blue stained sections for cartilage loss (0 = normal to 3 = severe) by two independent observers blinded to experimental groups^81^. Scores were summated, producing a mean arthritis index.

### Immunostaining for aggrecan neoepitopes

Primary antibodies for murine NITEGE and DIPEN were donated by John Mort (Shriners Hospital for Children, Montreal). Immunohistochemical staining for aggrecan breaks was performed using VECTASTAIN Elite ABC Kit (Mouse IgG) (Vectorlabs) following manufacturer’s instructions, with biotinylated goat anti-rabbit IgG secondary antibody and DAB (3,3′-diaminobenzidine; Sigma-Aldrich). Rehydrated sections underwent antigen retrieval in 1 mM EDTA at 65 °C overnight. Sections were blocked using 2.5% goat serum for 20 minutes prior to 30 minutes exposure to primary antibodies, rinsed with PBS and then exposed to biotinylated secondary antibody, ABC reagent and DAB before counterstaining with Mayer’s Haematoxylin, clearing and mounting. Mean counts were calculated for cells showing adjacent matrix stain for NITEGE or DIPEN in femoral and tibial cartilage (n = 11 (day 3), 9 (day 7), 4 (day 14) mice per group).

### Cytokine Quantification

IL-10 and TNFα in serum and CM-MSC were quantified using mouse Quantikine ELISA IL-10 immunoassay (R&D Systems) and TNFα ELISA high sensitivity (eBioscience) respectively, following manufacturer’s instructions.

### Detection of Tregs

Spleens from treatment and control animals sacrificed at 2 (n = 4) and 3 (n = 6) days post-arthritis induction were weighed and dissociated in PBS using a 7 ml borosilicate glass dounce tissue grinder with loose pestle (Fisherbrand). Cells were passed through a 30μm pre-separation MACS SmartStrainer (Miltenyi), centrifuged and resuspended in PBS pH 7.2 containing 0.5% bovine serum albumin and 2 mM EDTA. Tregs were isolated using CD4+ CD25+ Regulatory T Cell Isolation Kit (Miltenyi) following manufacturer’s instructions. CD4+ CD25+ cells were stained with CD4-FITC, CD25-APC and FOXP3-PE and analysed on a BD FACS CANTO II (BD Biosciences).

### Co-culture of T cells with MSC/CM-MSC

Proliferation of activated T cells was assessed as a measure of T cell deactivation. Initially, 5 × 10^4^ MSCs were cultured in 96-well plates for 24 hours at 37 °C. CD4+ T cells were purified from spleens and lymph nodes (popliteal/inguinal) of healthy C57Bl/6 mice using the CD4+ T Cell Isolation Kit (Miltenyi) following manufacturer’s instructions. T cells were seeded at a density of 5.0 × 10^5^/well in 250 μl RPMI medium with 10% FBS and MSCs (ratio 10:1) or with CM-MSC for 5 days. T cells alone served as control. Cells were activated using anti-Biotin MACSiBead Particles (Miltenyi) (ratio 2:1). Proliferation was assessed through reduction in signal intensity using VPD450 Violet proliferation dye (BD Biosciences) following previously described methodologies to calculate proliferative index and proliferative cycles^24,25^.

### Intracellular staining

Spleens and lymph nodes (popliteal/ inguinal) were collected from mice 3 days post-arthritis induction and dissociated as described. Splenocytes and lymph node cells were seeded separately at 1.0 × 10^6^ cells/well in 96-well plates (Sarstedt) in RPMI-1640 with 10% FBS, 0.05 μg/mL IL-2 and 10 μg/ml brefeldin A (Sigma) and activated with cell stimulation cocktail (eBioscience) at 37 °C for 4 hours. No cell stimulation cocktail and no brefeldin A served as controls. Following activation, cells were resuspended in 2 mM EDTA in PBS and Tregs (CD4+ CD25+ FOXP3+) were isolated using the CD4+ CD25+ Regulatory T Cell Isolation Kit (Miltenyi) following manufacturer’s instructions; or stained for T cell subset identification. For this, cells were permeabilised using permeabilisation buffer kit (eBioscience) and intracellularly stained with anti-mouse IFNγ (Th1), IL-4 (Th2) or IL17a (Th17) (eBioscience). Cells were analysed on a BD FACS Canto II flow cytometer and comparisons drawn for percentage CD4+ cells and signal intensity (XGeoMean) for each antibody.

### Statistical analysis

Data were tested for equal variance and normality using D’Agostino & Pearson omnibus normality test. Differences between groups were compared using 1-way ANOVA for parametric data or Kruskall-Wallis ANOVA for non-parametric, or 2 Way ANOVA with Bonferroni correction, as stated. All statistical analysis was carried out using Prism 5 (GraphPad software) or IBM SPSS Statistics 24.0, with P < 0.05 deemed statistically significant. Results are expressed as mean ± confidence interval. For regulatory T cell analysis, a reduction in cell size was observed following permeabilisation. Covariate of cell size (forward scatter, FSC-A) was used to compensate (ANCOVA).

### Data availability

All data generated or analysed during this study are included in this published article (and its Supplementary Information files).

## Electronic supplementary material

Supplementary Dataset 1
