# Supplementary material for: Mesenchymal Stem Cell-Conditioned Medium Reduces Disease Severity and Immune Responses in Inflammatory Arthritis
Source: Sci Rep. 2017 Dec 21;7:18019. doi: 10.1038/s41598-017-18144-w (PMC5740178; doi:10.1038/s41598-017-18144-w)
Supplement: Supplementary file 1 — Supplementary Dataset 1 [file 41598_2017_18144_MOESM1_ESM.doc]

## mesenchymal stem cell-conditioned medium reduces disease severity and immune responses in inflammatory arthritis.

Alasdair G. Kay^1,3^, Grace Long^2^, George Tyler^2^, Andrei Stefan^3^, Stephen J. Broadfoot^4^, Anna M. Piccinini^5^, Jim Middleton^6^, *Oksana Kehoe^3^

^1^Biology Department, University of York, Wentworth Way, York, UK ^2^School of Medicine, Keele University, Staffordshire, UK. ^3^ISTM at RJAH Orthopaedic Hospital, Keele University, Oswestry, UK. ^4^LSSU, Liverpool John Moore’s University, Liverpool, UK. ^5^School of Pharmacy, University of Nottingham, Nottingham, UK. ^6^Faculty of Health Sciences, School of Oral and Dental Science, University of Bristol, Bristol, UK

## *Correspondence and requests for materials should be addressed to O.K. (email: o.kehoe@keele.ac.uk)

## Supplementary data

### Supplementary tables

|  | **Day** | **Mean ± 95% CI** |
| --- | --- | --- |
| **Ctrl** | **1** | 0.047 ± 0.045 |
|  | **2** | 0.147 ± 0.069 |
|  | **3** | 0.400 ± 0.101 |
|  | **7** | 0.783 ± 0.213 |
|  | **14** | 0.900 ± 0.289 |
| **CM-treated** | **1** | 0.000 ± 0.000 |
|  | **2** | 0.352 ± 0.115 |
|  | **3** | 0.613 ± 0.134 |
|  | **7** | 1.000 ± 0.135 |
|  | **14** | 1.217 ± 0.234 |

**Table S1: Reduction in knee diameter from maximum for CM-MSC treated and SFM control knee joints (mm).**

|  |  | **NITEGE** | **DIPEN** |
| --- | --- | --- | --- |
|  | **Day** | **Mean ± 95% CI** | **Mean ± 95% CI** |
| **Ctrl** | **3** | 12.78 ± 7.90 | 19.71 ± 8.31 |
|  | **7** | 4.60 ± 6.22 | 9.28 ± 2.36 |
|  | **14** | 3.29 ± 19.56 | 9.09 ± 9.76 |
| **MSC-treated** | **3** | 2.41 ± 7.15 | 7.78 ± 10.22 |
|  | **7** | 4.32 ± 9.18 | 5.45 ± 6.66 |
|  | **14** | 0.54 ± 8.19 | 5.76 ± 14.38 |
| **CM-treated** | **3** | 0.70 ± 1.52 | -0.66 ± 3.26 |
|  | **7** | 3.57 ± 4.05 | 2.50 ± 5.83 |
|  | **14** | 10.95 ± 21.72 | 7.36 ± 16.43 |

**Table S2: Aggrecan breaks due to action of ADAMTS5 (early) and MMPs (late) contributing to cartilage degradation.**

|  | Days post-AIA induction | | |
| --- | --- | --- | --- |
|  | 3 | 7 | 14 |
| SFM | 5.31 | 12.86 | 23.56 |
| CM-MSC | 9.06 | 13.71 | 15.26 |

**Table S3: TNFα detection in serum of control (SFM) and CM-MSC treated mice.**

|  | **Proliferative Index** | **Proliferative Cycles/day** |
| --- | --- | --- |
| **Condition** | **Mean ± 95% CI** | **Mean ± 95% CI** |
| **T cell control** | 80.53 ± 1.65 | 1.185 ± 0.063 |
| **CM-MSC** | 79.67 ± 0.97 | 1.086 ± 0.109 |
| **MSC** | 76.14 ± 1.04 | 0.965 ± 0.071 |

**Table S4: Proliferative index for proportion of activated T cells proliferating alone, with CM-MSC or in co-culture with MSCs and complete proliferative 24 hour cycles over 5 days culture.**

|  |  |
| --- | --- |
| **Condition** | **Mean ± 95% CI** |
| **T cell alone** | 393.4 ± 196.8 |
| **CM-MSC** | 346.1 ± 321.5 |
| **MSC** | 117.9 ± 89.7 |

**Table S5: Co-culture medium tested for the presence of IL10 following 5 days culture with either activated T cells alone, in culture with CM-MSC, or co-culture with adhered MSCs.**
